# A single amino acid substitution confers B-cell clonogenic activity to the HIV-1 matrix protein p17

**DOI:** 10.1038/s41598-017-06848-y

**Published:** 2017-07-26

**Authors:** Cinzia Giagulli, Pasqualina D’Ursi, Wangxiao He, Simone Zorzan, Francesca Caccuri, Kristen Varney, Alessandro Orro, Stefania Marsico, Benoît Otjacques, Carlo Laudanna, Luciano Milanesi, Riccardo Dolcetti, Simona Fiorentini, Wuyuan Lu, Arnaldo Caruso

**Affiliations:** 1 Section of Microbiology, Department of Molecular and Translational Medicine, University of Brescia, 25123 Brescia, Italy; 2 Department of Biomedical Sciences, Institute for Biomedical Technologies e National Research Council (ITB-CNR), 20090 Segrate (MI), Italy; 3 Center for Translational Medicine, Xi’an Jiaotong University School of Life Science and Technology, Xi’an, Shaanxi 710048 China; 4 e-Science, Environmental Research and Innovation (ERIN) Department, Luxembourg Institute of Science & Technology (LIST), L-4422 Belvaux, Luxembourg; 5 Department of Biochemistry, University of Maryland School of Medicine, Baltimore, MD 21201 USA; 6 Department of Pharmacy, Health and Nutritional Sciences, University of Calabria, Arcavacata di Rende, 87036 Cosenza Italy; 7 Centre for Biomedical Computing, University of Verona, 37134 Verona, Italy; 8 Department of Pathology, University of Verona, 37134 Verona, Italy; 9 University of Queensland Diamantina Institute, Translational Research Institute, University of Queensland, Brisbane, QLD Australia; 10 Cancer Bio-Immunotherapy Unit, Centro di Riferimento Oncologico – IRCCS, Aviano, Italy; 11 Institute of Human Virology, University of Maryland, Baltimore, MD 21201 USA

## Abstract

Recent data highlight the presence, in HIV-1-seropositive patients with lymphoma, of p17 variants (vp17s) endowed with B-cell clonogenicity, suggesting a role of vp17s in lymphomagenesis. We investigated the mechanisms responsible for the functional disparity on B cells between a wild-type p17 (refp17) and a vp17 named S75X. Here, we show that a single Arginine (R) to Glycine (G) mutation at position 76 in the refp17 backbone (p17R76G), as in the S75X variant, is *per se* sufficient to confer a B-cell clonogenic potential to the viral protein and modulate, through activation of the PTEN/PI3K/Akt signaling pathway, different molecules involved in apoptosis inhibition (CASP-9, CASP-7, DFF-45, NPM, YWHAZ, Src, PAX2, MAPK8), cell cycle promotion and cancer progression (CDK1, CDK2, CDK8, CHEK1, CHEK2, GSK-3 beta, NPM, PAK1, PP2C-alpha). Moreover, the only R to G mutation at position 76 was found to strongly impact on protein folding and oligomerization by altering the hydrogen bond network. This generates a conformational shift in the p17 R76G mutant which enables a functional epitope(s), masked in refp17, to elicit B-cell growth-promoting signals after its interaction with a still unknown receptor(s). Our findings offer new opportunities to understand the molecular mechanisms accounting for the B-cell growth-promoting activity of vp17s.

## Introduction

HIV-1-associated lymphomas have not decreased after the introduction of combination antiretroviral therapy (cART) and non-Hodgkin’s lymphoma (NHL) represents the most common type of cancer^1, 2^ and the most frequent cause of death^3, 4^ in HIV-1-infected (HIV^+^) individuals. The HIV-1 genome is not integrated in the malignant B cells as seen for the oncogenic retrovirus HTLV-1. For this reason, the most shared assumptions rely on the indirect role of HIV-1 in lymphomagenesis. HIV-1-driven immune dysfunction with overproduction of B-cell stimulatory cytokines^5^ or loss of immune control, that promotes the reactivation of potentially oncogenic herpesviruses^6, 7^, are the most credited hypotheses up to date. However, novel findings support the possibility that HIV-1 may directly contribute to lymphomagenesis through mechanisms involving the biologic effects mediated by its gene products.

The HIV-1 matrix protein p17 (p17) is a 132 amino acid (aa)-long structural protein, composed of five major α-helixes and a highly basic platform consisting of three β strands^8, 9^. Four helixes are centrally organized to form a compact globular domain and a fifth helix (H5) in the COOH-terminus is slightly destabilized, due to the flexible C-terminal tail^10, 11^. The matrix protein is continuously released in the extracellular space from HIV-1-infected cells^12^. It has been detected in the plasma^13^ and in tissue specimens, such as brain^14^, liver^15^, bone marrow^16^ and lymph nodes of HIV^+^ patients^17^. In lymph nodes p17 accumulates and persists even during cART and in the absence of any HIV-1 replicative activity^17^, thus suggesting that it may be persistently expressed in the tissue microenvironment, even during pharmacological therapy, and promote chronic B-cell stimulation^18, 19^.

Latest data show that p17 expression in mice transgenic for a defective HIV-1 provirus is associated with lymphoma development^20, 21^. Moreover, p17 variants (vp17s), characterized by scattered mutation along the entire protein sequence^22^ or by specific aa insertions in the C-terminal region, display a potent B-cell growth-promoting activity triggering the PTEN/PI3K/Akt pathway^22, 23^, which is known to be crucial in lymphoma development^24^. Therefore, specific mutations within refp17 could induce a different pathogenetic potential to the viral protein. All these findings call for defining the structure-function relationship in clonogenic vp17s as compared to their wild-type counterpart.

In this study, we investigated the aa substitutions, the structural bases and the molecular mechanisms responsible for opposite effects in modulating B-cell growth between a vp17 derived from a Ugandan HIV-1 strain (subtype A1), named S75X^22^, and the wild type p17 (reference p17, refp17; from clone BH10 of the clade B isolate).

Here, we demonstrate that a single arginine (R) to glycine (G) mutation at position 76 in the refp17 backbone, as in S75X, is sufficient to induce dramatic changes in protein folding and stability, making p17 mutant capable of activating Akt and promoting B-cell proliferation.

## Results

### The R76G mutation in the refp17 backbone induces changes in the protein secondary structure and hydrogen bond network

**Figure 1:**
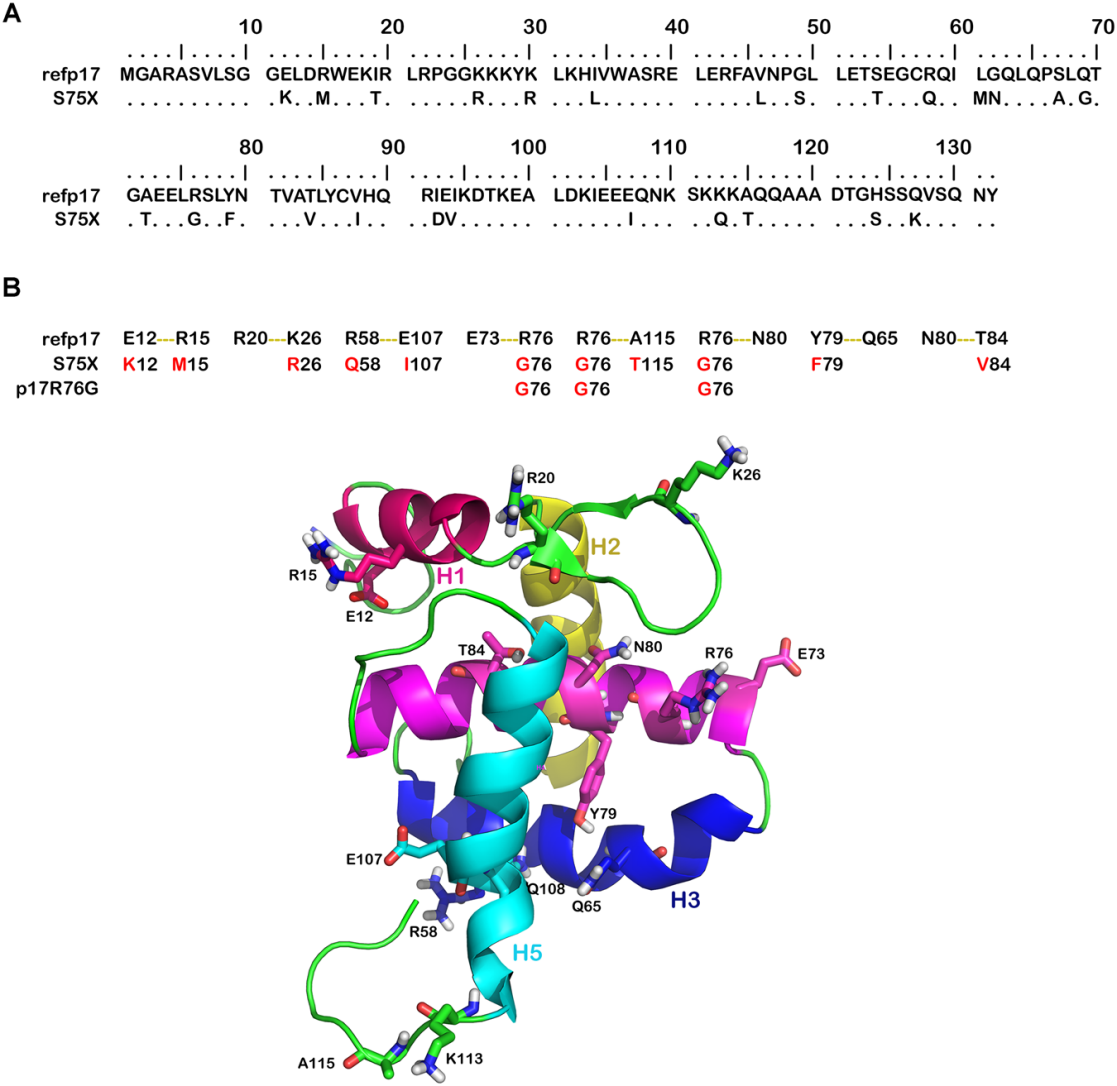
Sequence and hydrogen bond network of refp17 and S75X. (**A**) Sequences are represented by the single-letter amino acid code. The amino acid sequence (aa 1–132) of the matrix protein clade B isolate BH10 p17 (UniProtKB P04585) was adopted as reference (refp17) for this analysis. Each amino acid residue of S75X not differing from refp17 sequence is represented by a dot. (**B**) At the top of the Figure, in the first row, the residues involved in the hydrogen bond network of p17 are shown and below the residues mutated in the variants S75X and p17R76G are indicated. The residues involved in hydrogen bond network are shown as sticks.

We performed *in silico* studies to elucidate if aa mutations in S75X were responsible for changes in folding and stability of the viral protein as compared to refp17. Since mutated residues in S75X (Fig. 1A) are donors and acceptors of hydrogen bonds, we first performed long Molecular Dynamics simulation (MD) (500 ns) of refp17 to evaluate its hydrogen bond network. Side chains of mutated residues in S75X and involved in hydrogen bonds are shown in Fig. 1B. In order to identify key residues involved in interaction network, we evaluated hydrogen bonds during the entire refp17 MD and set the threshold of essential hydrogen bonds in the frames of all the trajectory to 75%^25^. The identified hydrogen bonds were E73-R76 (88%), N80-T84 (83%) and R58-E107 (81%) and the identification of E73-R76 as the most frequent hydrogen bond suggested us to further investigate its role in protein structure. In S75X the residue R76 results mutated in a G and this aa substitution leads to the loss of the E73-R76 hydrogen bond. Therefore, in order to investigate the role of this hydrogen bond in the stability and folding of the matrix protein, we modeled S75X and p17R76G, a p17 mutant with R76 replaced by a G, and performed long MD (500 ns).

The stability of refp17, S75X and p17R76G was defined on the basis of these conditions along the dynamics: the total energy less than −40000 kcal/mol, RMSD of the structure in a range of 1 Angstrom around the centre of oscillations, the formation of hydrogen bonds, and the size of nonpolar surface area. Refp17 showed a higher stability than S75X and p17R76G, exhibiting a higher average number of hydrogen bonds (58 versus 48.5 and 55, respectively) and lower nonpolar surface areas (31.3% versus 33, 87% and 34, 23%, respectively).

In addition, the MD frames of p17s were clustered (% of the frames): two clusters (42% and 43%) in refp17, five clusters (29%, 26%, 15%, 9% and 7%) in the S75X and three clusters (25%, 23% and 22%) in p17R76G were obtained. The dispersion indices were 12.39, 31.65 and 20.00 for refp17, S75X and p17R76G, respectively. All these results suggest that S75X and p17R76G are less structured as compared to refp17.

**Figure 2:**
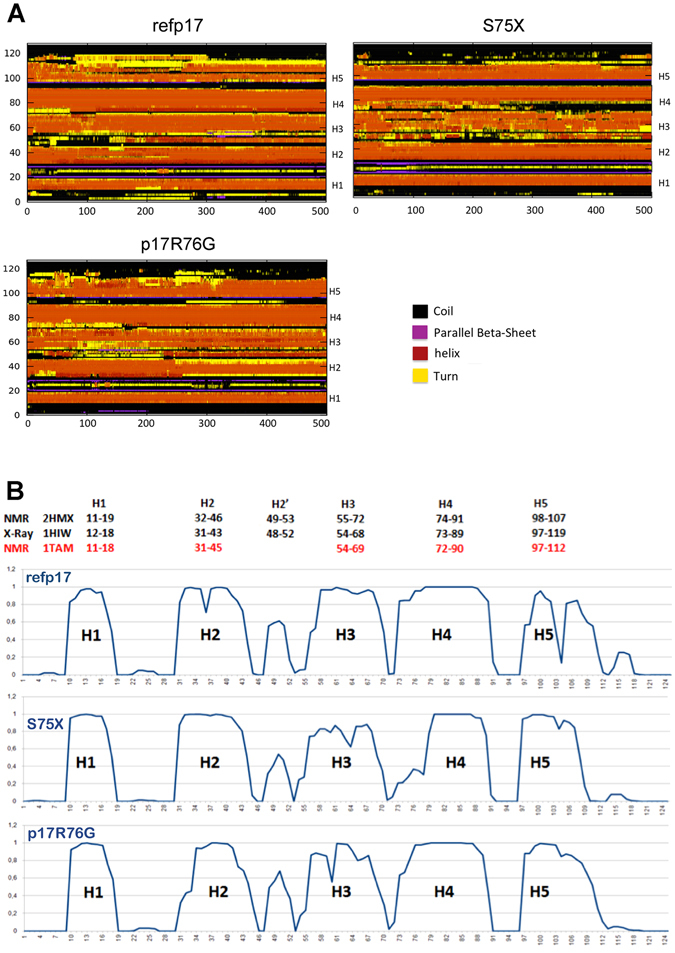
Evolution of refp17, S75X and p17R76G protein structure by molecular dynamics simulations. (**A**) Time evolution of the secondary structural elements along the molecular dynamics simulation generated by DSSP. The X-axis represents the molecular dynamics trajectory time (in ns), while the residue numbers are shown on the Y-axis. (**B**) At the top of the Figure are indicated the residues involved in forming the helix structures of refp17 and determined experimentally by NMR (PDB code: 2HMX and 1TAM) and X-Ray (PDB code: 1HIW). In the graph the propensity for each amino acid residue to assume helix structure calculated during simulations of refp17 and its variants.

In addition, using the DSSP software and calculating the average of helix propensities of each aa along the trajectory, we assessed whether mutations in S75X and p17R76G induce any changes in secondary structure^26^. Interestingly, S75X and p17R76G showed a disorganization in the N-terminal region of H3 and H4, and in the C-terminal region of H3 (Fig. 2). This was not observed in refp17, whose helices of globular domain were stable during simulation. A greater stability of H5 is observed in S75X and p17R76G as compared to refp17 (Fig. 2). The stability of H5 in S75X is due to the formation of the new hydrogen bond R91-E106 and in p17R76G of the R58-Q108 and Y86-E107 bonds. On the contrary, after 140 ns of MD in refp17 we observed a break in the middle of H5 (residue 105) resulting in displacement of structured C-terminal region towards the N-terminal region of H3. This shift allows the formation of a hydrogen bond R76-A112, obtaining a new interaction region. In all three proteins, fluctuations from helix to turn or coil structures were observed in the C-terminal region of H5 (Fig. 2).

Overall, these findings show that changes in the hydrogen bond network, due to the insurgence of even a single point mutation (R76G), are responsible for changes in the secondary structure of refp17.

### The R76G mutation destabilizes refp17 and alters its ability to oligomerize in solution

**Figure 3:**
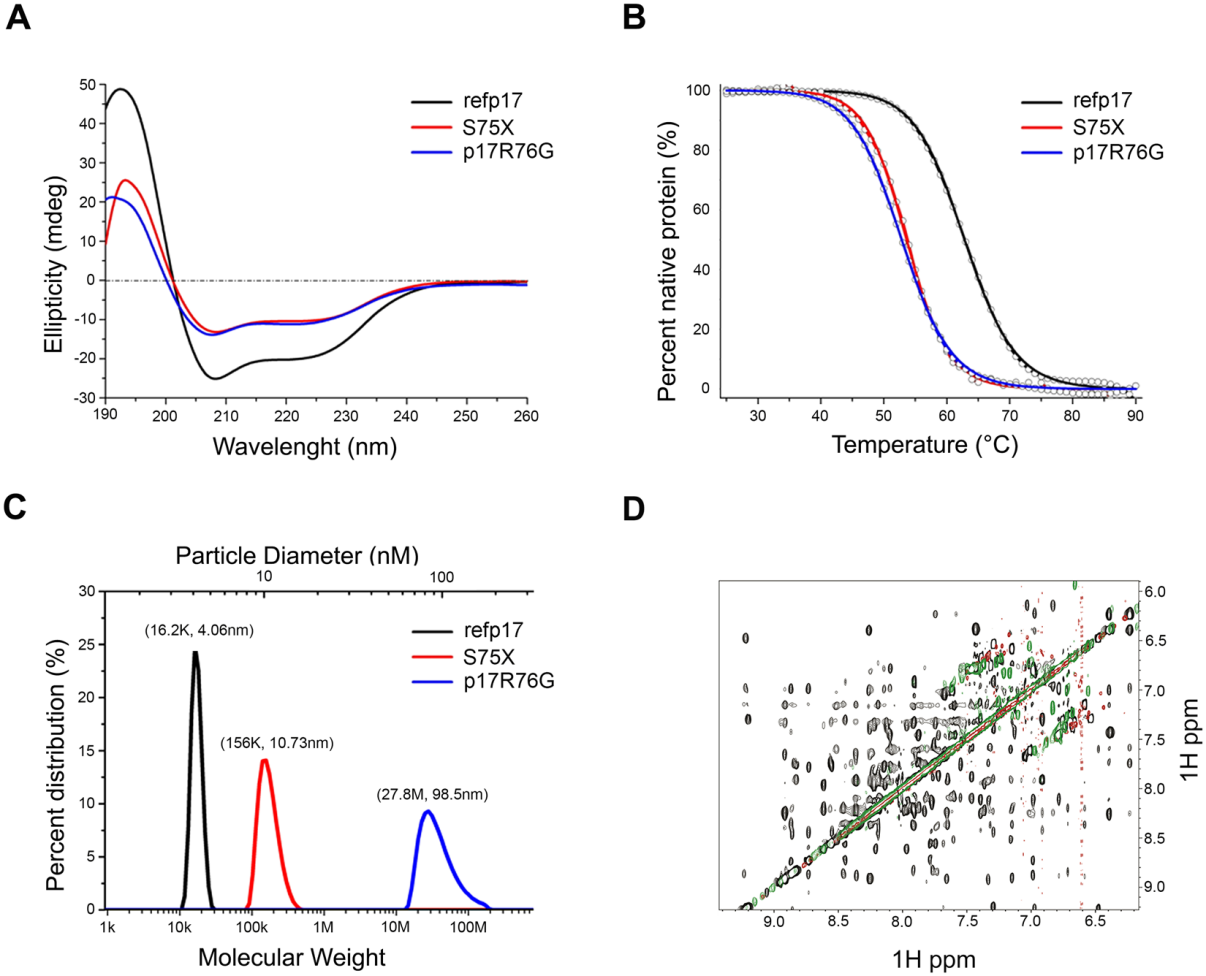
Structure and stability of refp17, S75X and p17R76G. (**A**) CD spectra of refp17, S75X, and p17R76G collected at room temperature at 2.5 μM in 10 mM phosphate buffer, pH 7.4. (**B**) Thermal denaturation of refp17, S75X, and p17R76G at 10 μM in PBS, pH 7.4, as monitored at 222 nm by CD spectroscopy. The experimental data were normalized according to a two-state protein denaturation model. Note that the thermal denaturation of p17 is irreversible due to protein aggregation. (**C**) Measurement of the size of folded refp17, S75X and p17R76G viral matrix proteins by the technique of dynamic light scattering at room temperature. (**D**) NMR spectra of refp17 (black), S75X (green), and p17R76G (red) showing their amide-amide NOEs.

In order to confirm conformational shifts evidenced by MD, the structural conformation of refp17, S75X and p17R76G was investigated. To this aim we generated the three recombinant viral proteins and analyzed them in aqueous solution (each at 2.5 μM), using circular dichroism (CD) spectroscopy. All proteins displayed alpha-helical secondary structures at room temperature, as evidenced by a strong positive maximum at 195 nm and two negative minima at 208 and 222 nm (Fig. 3A), consistent with the known structural features of p17^8, 9^. However, S75X and p17R76G were noticeably less helical than refp17, as indicated by the reduction in ellipticity, suggesting a partial unfold of proteins (Fig. 3A). In order to evaluate the impact of mutations on protein structure, we subjected refp17, S75X and p17R76G to heat-induced denaturation and monitored them at 222 nm by CD spectroscopy. As temperature was raised from 25 °C to 90 °C, all three proteins exhibited a co-operative unfolding with a single transition, typical of a compact globular protein (Fig. 3B). Data normalization based on a two-state protein denaturation model gave rise to a characteristic melting temperature (Tm at which 50% of protein is denatured or unfolded) for each protein. S75X and p17R76G had similar Tm values, approximately 10 °C lower than refp17 (Table 1). These results show that mutations in vp17s destabilize the matrix protein, corroborating findings from CD spectroscopy.

**Table 1:** Values of Tm and ΔHm determined through thermal denaturation at pH 7.4 and protein size measured by dynamic light scattering.

	refp17	S75X	p17R76G
T_m_ (°C)	62.82 ± 0.09	53.67 ± 0.15	52.83 ± 0.04
∆H_m_ (kcal/mol)	56.13 ± 0.91	63.80 ± 2.23	53.75 ± 0.43
Nominal molecular weight (Da)	15026	14864	14926
Particle molecular weight (Da)	16.2K	156K	27.8M
Granule diameter (nm)	4.06	10.7	98.5
Aggregation form	Monomer	Multimer	Multimer

Since temperature-induced unfolding is usually accompanied by protein aggregation^27^, we examined the ability of vp17s to oligomerize in solution by dynamic light scattering techniques (Table 1). In solution refp17 exists as monomer or trimer depending on protein concentration^28^. At 10 μM refp17 existed as a monomer (Fig. 3C), while S75X and p17R76G displayed a molecular weight 10 and 170 fold higher than refp17 (Fig. 3C), respectively. These data suggest that a single aa mutation occurring in the refp17 backbone can induce a massive self-association, indicating that some refp17 buried hydrophobic residues become exposed in S75X and p17R76G.

Then, we analyzed the three p17 proteins using 2-dimensional NMR spectroscopy and their amide-amide Nuclear Overhauser Effect Spectroscopy (NOESY) spectra. As shown in Fig. 3D, refp17 displayed a large number of well-dispersed amide-amide Nuclear Overhauser Effect (NOE) signals, which are characteristic of a well-folded alpha-helical protein. On the contrary, S75X and p17R76G showed very few amide-amide NOE signals in the same region of the spectrum, consistent with dampened signals arising from the formation of soluble aggregates.

Overall, our data suggest that a single R76G mutation in refp17 backbone can destabilize the viral protein with a reduction of alpha-helical secondary structures, thermal stability and soluble aggregates formation.

### S75X and p17R76G promote B-cell growth

**Figure 4:**
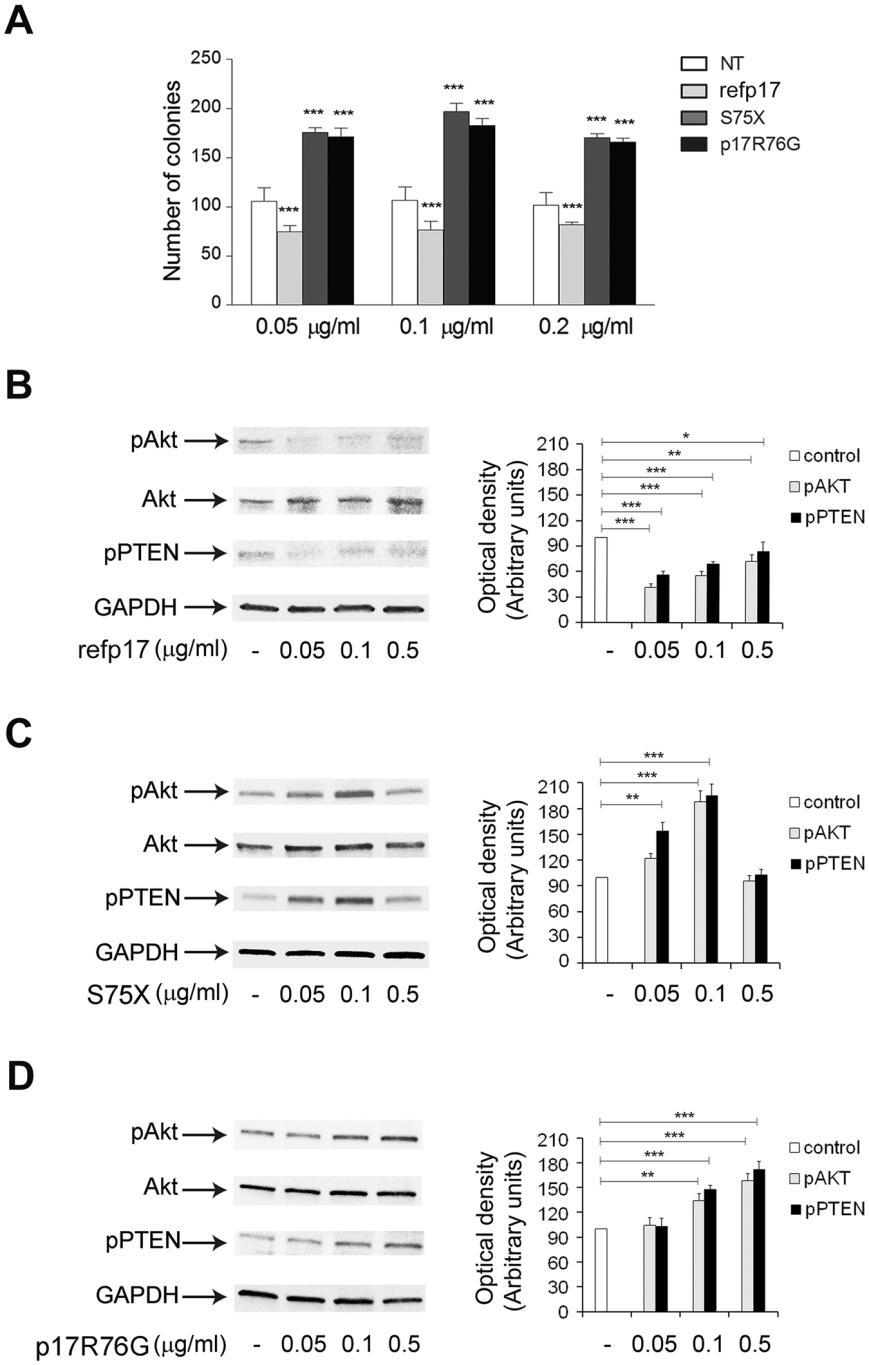
Effect of refp17, S75X and p17R76G on B-cell activity. (**A**) Raji were plated in twelve-well plates and, after four days, medium was replaced by fresh medium with the indicated concentration of refp17, S75X and p17R76G. Cells not treated (NT) were used as negative control. The cell growth was analyzed by using MTT. Data represent the average number of colonies ± SD from three independent experiments performed in triplicate. The statistical significance between control and treated cultures was calculated using one-way ANOVA performed separately for each concentration of p17 variants and Bonferroni’s post-test was used to compare data; ***P < 0.001. (**B**–**D**) Cells were treated for 5 min with 0.05, 0.1, 0.5 μg/ml of refp17 (**B**), S75X (**C**) and p17R76G (**D**). Untreated cells were used as control. Western blot analysis of Raji lysates shows that refp17 inhibits the activation of Akt and maintains PTEN in an active state (**B**), as shown by the respective phosphorylation state at any concentration tested, verified by densitometric analysis and plotting of the pAkt/Akt and pPTEN/GAPDH. On the contrary, either S75X or p17R76G induce the activation of Akt and maintains PTEN in an inactive state (**C**,**D**), as shown by the increased phosphorylation, verified by densitometric analysis and plotting of the pAkt/Akt and pPTEN/GAPDH. In the left panel blots from one representative experiment of three with similar results are shown. In the right panels, values reported for phosphorylation of Akt and PTEN are the mean ± SD of three independent experiments. Statistical analysis was performed by one-way ANOVA and the Bonferroni’s post-test was used to compare data; *P < 0.05; **P < 0.01; ***P < 0.001.

Destabilization of refp17 with changes in the secondary structure, induced by aa mutations, are known to confer conformational changes sufficient to endow the viral protein with B-cell growth-promoting activity^23^. Since in refp17 the E73-R76 hydrogen bond was identified by MD as an essential interaction for hydrogen bond network and CD and NMR spectroscopy confirm a destabilization of viral protein (Fig. 3), we aimed at determining whether p17R76G, a mutant bearing a G at position 76 instead of an R, as in S75X, could acquire a B-cell clonogenic activity. Then, we tested the p17R76G capability of modulating B-cell growth by soft agar clonogenic assays. As shown in Fig. 4A, refp17 inhibited the colony-forming ability of Raji B cells as compared to untreated cultures, while S75X and p17R76G enhanced their clonogenic activity. These results confirm that the rupture of the E73-R76 hydrogen bond generates significant structural changes conferring to the matrix protein a S75X-like B-cell clonogenic activity.

### S75X and p17R76G activate the PTEN/PI3K/Akt pathway in B cells

The PTEN/PI3K/Akt pathway represents the major intracellular signaling cascade known to regulate proliferation and malignant transformation^29, 30^. Refp17 was shown to enhance PTEN activity and down-modulate the PI3K/Akt signaling pathway, exerting an anti-proliferative effect on B cells, while S75X was shown to block PTEN and activate Akt, thus promoting B-cell growth^22^. Due to clonogenic activity of p17R76G (Fig. 4A), we explored its capability to modulate the PTEN/PI3K/Akt pathway. As shown in Fig. 4B, Raji cells stimulated with refp17 showed a significant Akt inhibition, as expected, while S75X and p17R76G phosphorylated Akt kinase (Fig. 4C and D). Since Akt can be modulated by PTEN^30^, we also evaluate the Ser/Thr phosphorylated PTEN inactivation form. As shown in Fig. 4C and D, cells treated with S75X and p17R76G showed an increased PTEN phosphorylation as compared to unstimulated cells, whereas treatment of cells with refp17 resulted in a reduced PTEN phosphorylation level (Fig. 4B). Overall, these data show that p17R76G, like S75X, activates the proliferative PI3K/Akt pathway in B cells through PTEN down-modulation.

### S75X and p17R76G modulate different signaling molecules involved in cell cycle regulation

Assessment of protein phosphorylations is essential for understanding intracellular activities controlling cell functions. To further investigate the intracellular mechanisms involved in the B-cell growth-promoting activity of S75X and p17R76G, we assessed how these proteins modulate intracellular phosphorylations by employing a high-throughput antibody array technology. The lysates of Raji cells, stimulated or not with refp17, p17R76G or S75X (0.1 μg/ml) were processed by Kinexus Bioinformatics Corporation for Kinex™ KAM-850 Antibody Microarray. Then, in order to rule out false positive array signals, all the protein modulated by S75X and p17R76G were confirmed by a multi-immunoblotting analysis performed by Kinexus. The signals relative to the 23 proteins modulated by S75X and p17R76G, verified by densitometric analysis and normalized to refp17, are reported in Table 2. Literature data mining and GO biological process database highlighted that most of modulated proteins, as expected, are involved in cell cycle regulation (CDK1, CDK2, CDK8, CHEK1, CHEK2, GSK-3 beta, NPM, PAK1, PP2C-alpha) and apoptosis (CASP-9, CASP-7, DFF-45, NPM, YWHAZ, Src, PAX2, MAPK8).

**Table 2:** Effect of S75X and p17R76G on expression and phosphorylation levels of intracellular signaling proteins.

Protein short name	Uniprot	Protein name	Phosphorylation site	S75X vs refp17 (log)	p17R76G vs refp17 (log)	S75X vs refp17 (FC)	p17R76G vs refp17 (FC)
YWHAZ	P63104	14-3-3 protein zeta/delta	—	0, 26	0, 12	1, 20	1, 09
NPM	P06748	Nucleophosmin	T199	0, 20	1, 15	1, 15	2, 22
CaMKK2	Q96RR4	Calcium/calmodulin-dependent protein kinase kinase 2	—	0, 52	0, 50	1, 44	1, 41
CASP-7	P55210	Caspase-7	—	0, 94	0, 12	1, 92	1, 09
CASP-9	P55211	Caspase-9	—	0, 43	0, 38	1, 35	1, 31
CDK1/2	P24941	Cyclin-dependent kinase 1/2	Y15	−0, 28	−0, 41	−1, 21	−1, 32
CDK1/2	P24941	Cyclin-dependent kinase 1/2	T14 + Y15	−0, 17	−0, 06	−1, 12	−1, 04
CDK2	P24941	Cyclin-dependent kinase 2	—	0, 37	0, 42	1, 30	1, 34
CDK8	P49336	Cyclin-dependent kinase 8	—	0, 36	0, 22	1, 28	1, 17
CHEK1	O14757	Serine/threonine-protein kinase Chk1	—	0, 87	0, 81	1, 82	1, 75
CHEK2	O96017	Serine/threonine-protein kinase Chk2	T68	0, 08	0, 26	1, 06	1, 20
DFF-45	O00273	DNA fragmentation factor subunit alpha	—	0, 41	0, 85	1, 32	1, 80
GSK-3 beta	P49841	Glycogen synthase kinase-3 beta	Y216	0, 34	0, 27	1, 26	1, 20
MAPK8	P45983	Mitogen-activated protein kinase 8	—	0, 17	0, 23	1, 12	1, 18
Lyn	P07948	Tyrosine-protein kinase Lyn	Y508	−1, 46	−0, 08	−2, 76	−1, 06
PAK1	Q13153	Serine/threonine-protein kinase PAK1	T212	1, 31	0, 58	2, 48	1, 50
PAX2	Q02962	Paired box protein Pax-2	S394	1, 25	0, 38	2, 38	1, 30
PKC-B	P05771	Protein kinase C beta type	—	0, 27	0, 60	1, 20	1, 52
PP2C-alpha	P35813	Protein phosphatase 1A	—	0, 67	1, 55	1, 59	2, 92
PP2C-delta	O15297	Protein phosphatase 1D	—	0, 69	1, 69	1, 61	3, 23
PP5	P53041	Serine/threonine-protein phosphatase 5	—	0, 12	0, 48	1, 09	1, 40
SOCS-4	Q8WXH5	Suppressor of cytokine signaling 4	—	−0, 60	−0, 28	−1, 52	−1, 21
Src	P12931	Src proto-oncogene-encoded protein-tyrosine kinase	—	−0, 19	−0, 55	−1, 14	−1, 47

The protein expression and phosphorylation level were analyzed from Kinexus Bioinformatic Corporation by densitometric analysis of multi-immunoblotting. Blot raw quantification data were processed by log transformation and normalization to either not treated or p17 treated samples for the interpretation of the results. The numerical values refer to log2-ratio with respect to refp17 values. FC = Fold Change.

**Figure 5:**
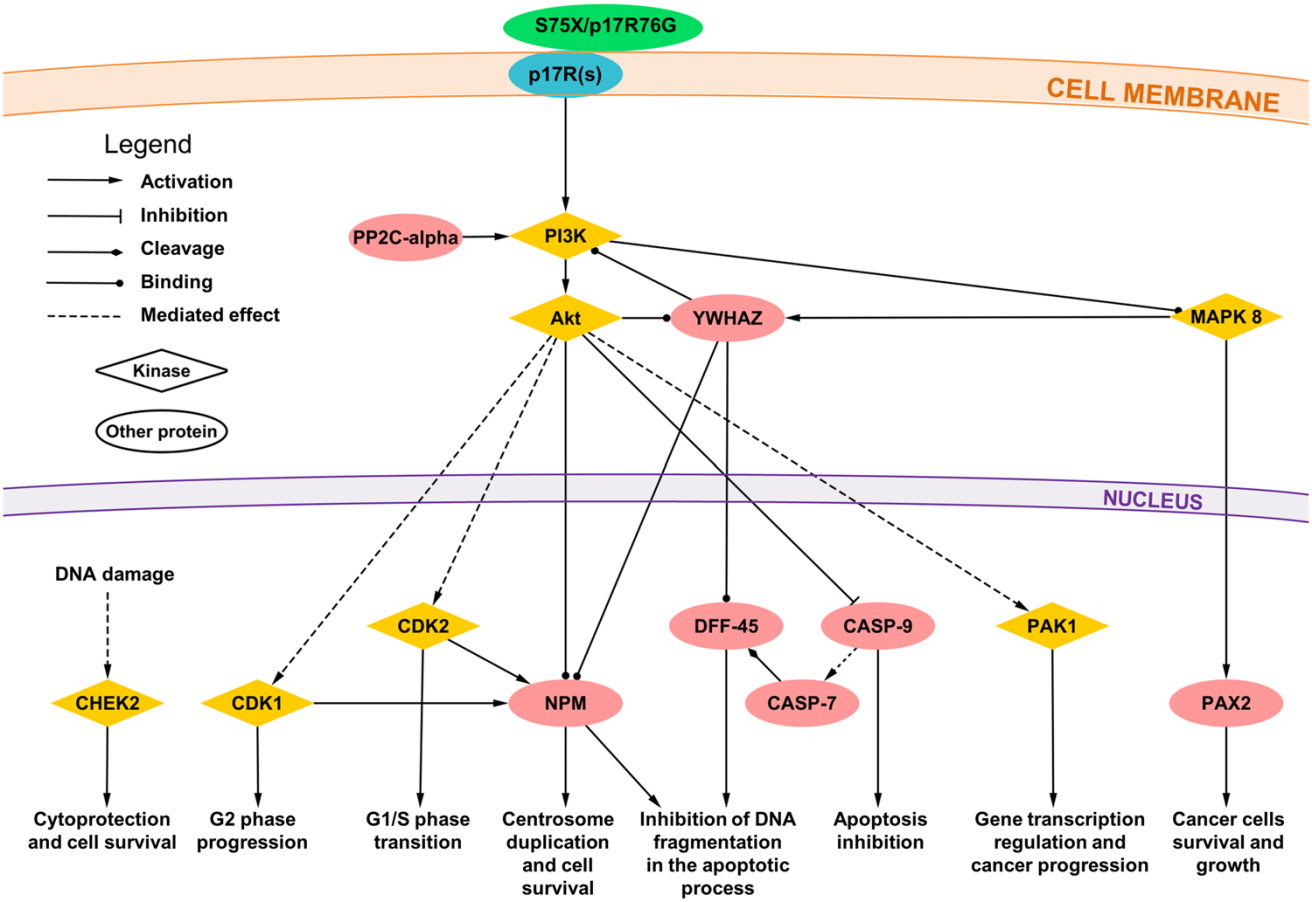
Representation of the putative signaling pathways involved in B-cell clonogenicity induced by interaction of S75X/p17R76G with p17R(s). Stimulation of B cells with the clonogenic p17 proteins, S75X and p17R76G, induces the activation of several molecules involved in promoting cell survival, cell cycle progression and in inhibiting apoptosis, and STRING database and literature data mining were used to identify known and experimentally verified interactions. The several kinases involved in the pathway are indicated by orange diamonds, the other proteins are represented by light red ellipses. PI3K: phosphatidylinositol-3-kinase; Akt: Protein kinase B;PP2C-alpha: Protein phosphatase 1A; MAPK8: Mitogen-activated protein kinase 8; YWHAZ: 14-3-3 protein zeta/delta; CHEK2: Checkpoint kinase 2; CDK1: Cyclin-dependent kinase 1; CDK2: Cyclin-dependent kinase 2; NPM: Nucleophosmin; DFF-45: DNA fragmentation factor subunit alpha; CASP-9: caspase-9; CASP-7: caspase-7; PAK1: Serine/threonine-protein kinase PAK 1; PAX2: Paired box protein Pax-2.

In Fig. 5 we established the known and experimentally verified molecular interactions among the proteins reported in Table 2 using STRING database^31^ of Protein-Protein Interaction Network and literature data mining. Those molecules not displaying confirmed interactions (CaMKK2, CDK8, Lyn, PP2C-delta, SOCS-4, Src, CHEK1, GSK-3 beta, PP5, PKC-beta) were kept out from the signaling network, even if we cannot completely rule out their involvement in vp17s-induced B-cell growth. As shown in Fig. 5, S75X and p17R76G trigger the PI3K/Akt signaling pathway, which in turn could activate MAPK8^32^ and YWHAZ^33^, thus creating a positive feedback loop further strengthening Akt activation^34^. In addition, MAPK8, besides its ability to activate PAX2 and promote cancer cell proliferation^35^, can trigger YWHAZ and inhibit DNA fragmentation process during apoptosis by interacting with DFF-45^36^. PP2C-alpha up-regulation can also concurs likely to further activate PI3K/Akt pathway^37^. Moreover, Akt activation can modulate the activity of PAK1^38, 39^, CASP-9^40^, DFF-45^36, 41^, NPM^42, 43^, CDK1 and CDK2^44, 45^. Among these factors, a central role can be exerted by NPM, which is known to be directly activated by Akt, but also by YWHAZ and CDK1/2^46–48^. Finally, CHEK2 activation can be involved in cytoprotection and cell survival^49^.

These data highlight that the activation of PI3K/Akt pathway in B cells by clonogenic S75X and p17R76G can orchestrate the function of several molecules involved in promoting tumor survival and proliferation.

## Discussion

The function of a globular protein is governed by its 3D structure, while different biological functions can be performed by a protein that lacks the ordered secondary structure and thermal stability^50–52^. As several cancer-associated proteins, p17 possesses high levels of predicted intrinsic disorders^53, 54^, which subtend to a partially unfolded status of specific regions. Previously, we showed that in S75X, which contains aa mutations scattered throughout the p17 protein sequence, specific hydrogen bonds stabilizing the refp17 structure are lost, giving rise to a partially unfolded - B-cell growth-promoting - protein^18^. However, it is known that different aa residues can be mutated in vp17s without loss or change in their refp17-like functional activity, suggesting that the clonogenic activity of vp17s do not depend from protein sequence, but only from specific mutations capable of endowing p17s with a B-cell growth-promoting activity^23^. MD simulations showed that S75X displays a single aa mutation (R76G) that is likely to be the most responsible for protein destabilization, with reduction of alpha-helical secondary structures and for shifting the equilibrium toward a partially unfolded conformation. CD and 2-dimensional NMR spectroscopy confirmed this prediction using a recombinant p17 mutant carrying the single R76G replacement in the refp17 backbone (p17R76G). As expected, clonogenic assays showed that the replacement of R to G at position 76, as in S75X, is sufficient to endow refp17 with a B cells growth-promoting activity. Therefore, aa mutations, which alter the hydrogen bond network and contribute to an even partial unfolding process of refp17, confer to vp17s different functional outcomes compared to refp17.

Unfolding and subsequent self-assembly of proteins into various aggregates are common molecular mechanisms involved in important human diseases^55, 56^. Our data show that the partially unfolded conformational ensemble of vp17s favors specific intermolecular interactions and protein aggregation. Indeed, vp17s exhibit higher nonpolar surface areas than refp17, probably due to the exposure of some buried hydrophobic residues, which induces them to massively self-aggregate. Further studies will address if some of the p17 cell toxic activities^57^ are dependent on this aggregation status.

The p17R76G protein, similarly to S75X^22^, was also found to activate Akt kinase, modulating PTEN activity. High-throughput phosphoproteomic and bioinformatics analysis highlighted a central role of Akt kinase in the proliferative signaling pathways induced by S75X and p17R76G in B cells. Indeed, Akt can modulate the function of several molecules as PP2C-alpha, YWHAZ, MAPK8, PAX2, PAK1, CASP-9, CASP-7, DFF-45, NPM, PAX2, CDK1, CDK2, CHEK2, involved in apoptosis inhibiton, cell cycle promotion and cancer progression. All these findings were not unexpected since the PTEN/PI3K/Akt pathway represents the major signaling cascade known to play a central role in tumorigenesis^58, 59^.

**Figure 6:**
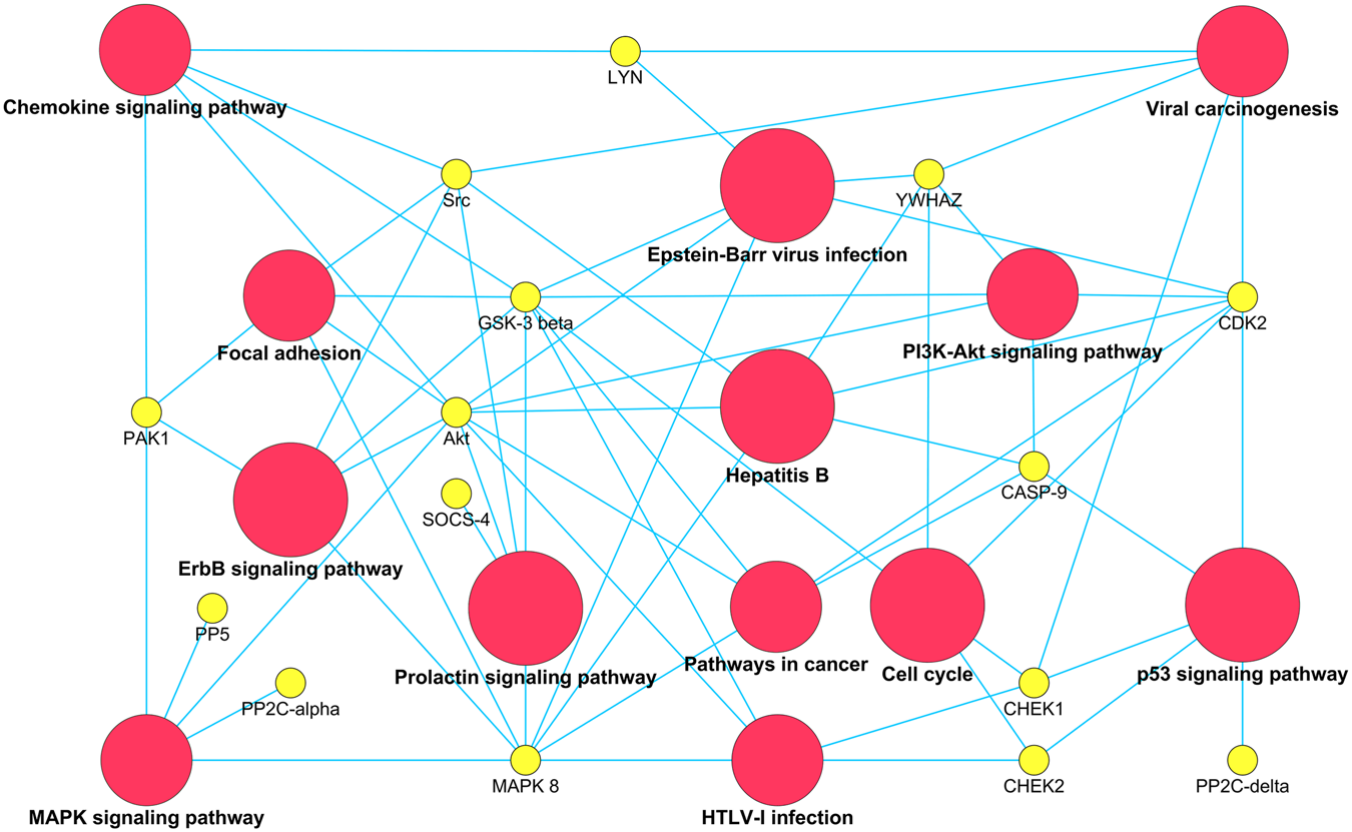
Representation of KEGG pathways significantly regulated by S75X and p17R76G. The proteins modulated by p17s were used as input for the Cytoscape ClueGO functional analysis tool to cluster the proteins according to the KEGG pathways database. The proteins are represented by yellow circles. The pathways are represented by red circles and the circle size is directly proportional to statistical relevance (3 * 10^−3^ > p < 8 * 10^−6^). An edge is present when the protein is involved in the pathway.

All molecules modulated by S75X and p17R76G, used as input for the Cytoscape ClueGO functional analysis tool^60^ to cluster them according to the KEGG biological pathway, result to be related to several known biological pathways. As shown in Fig. 6, most of the identified signaling pathways are related to cancer development and progression, as PI3K/Akt, p53, MAPK and ErbB. It is important to underline that molecules modulated by S75X and p17R76G are also involved in signaling triggered by oncogenic viruses, like EBV, HTLV-1 and HBV. This latter observation suggests that different viruses, and among them HIV-1, may exploit common paths to promote cell proliferation. Moreover, EBV infection was recently shown to up-regulate p17Rs expression on primary B lymphocytes, thereby making these cells permissive to the biological effects mediated by p17s^19^. Interestingly, molecules modulated by S75X and p17R76G were also related to the prolactin signaling pathway, supporting previous data showing the presence of higher serum prolactin in HIV^+^ patients than in healthy individuals and the capability of prolactin to influence normal lymphocyte mitogenesis and lymphoid tumor growth^61–63^. Finally, some molecules modulated by clonogenic vp17s result to be related to chemokine signaling pathway and this finding is not surprising, since p17 is known to act as a viral chemokine^15, 64–66^.

Recently, we showed that vp17s derived from HIV^+^ NHL patients were characterized by specific aa insertions in the C-terminal region, tempting to ascribe this activity to the C-terminus of the protein interacting directly with its putative receptors^23^. Nevertheless, we previously described that the presence of H5 in p17 is not necessary for protein clonogenic activity^22^, highlighting the intrinsic capability of p17 to exert B-cell clonogenic activity following protein unfolding. Indeed, truncation of the last 36 aa residues of p17 resulted in a protein (p17Δ36) capable of promoting B-cell clonogenicity through PTEN/PI3K/Akt pathway. Moreover, the different biological activity of S75X and p17R76G compared to refp17, underlines how the matrix protein binding to and signaling through p17R(s) can potentially involve distinct determinants of structure or conformations. For these reasons, we hypothesize that a specific clonogenic epitope responsible for B-cell clonogenic activity, located probably in N-terminal or in the core of matrix protein, is masked in the properly folded monomeric p17, but exposed in misfolded, aggregated B-cell vp17s. Therefore, the molecular reasons for the opposite mechanisms among mutated proteins and wild-type p17 may depend on the exposure or not of a clonogenic epitope and from the activation of the PI3K/Akt pathway, which is specific for the mutated clonogenic proteins. This downstream signaling pathway might be ascribed to the interaction of the viral mutated proteins, in their unfolded or aggregated form, with a different p17R(s) from the ones known engaged by refp17. Up to date, the direct interactor of clonogenic mutated proteins is still unknown and its identification will be sure matter of further investigations.

In conclusion, our study represents the first molecular and structural characterization of a single point mutated p17 displaying B-cell clonogenic activity. Our findings open the way for a better understanding of the structure-function relationship underlying the B-cell clonogenic activity of some vp17s. Moreover, demonstration that clonogenic vp17s exert their action on B cells through activation of the PTEN/PI3K/Akt pathway, which represents a critical driver of lymphoma development and metastasis, may offer new opportunities to identify novel treatment strategies in combating HIV-related B-cell lymphoma. Finally, our results provide the rational background for further studies aimed to assess the possible molecular signatures identifying vp17s with B-cell clonogenic activity and recognition of HIV^+^ individuals at risk to develop lymphoma.

## Materials and Methods

### Cell cultures

Human lymphoblastoid cell line (Raji) was obtained from the American Type Culture Collection (ATCC) and cultured in RPMI-1640 medium containing 10% fetal calf serum, 1 mM L-glutamine and 1mM sodium pyruvate.

### Recombinant proteins

Purified endotoxin-free recombinant HIV-1 matrix protein p17, S75X and p17R76G were produced as previously described^22^. The mutated matrix protein p17R76G, in which the arginine in position 76 was replaced by a glycine, was generated from p17 sequence by using Quick Change Site-directed mutagenesis Kit (Stratagene).

### Molecular Modeling

The structures of refp17 were obtained from the Protein Data Bank (PDB) code 1TAM, whereas homology modeling (Modeler) was used to obtain the structures of S75X and p17R76G. The PDB code 1TAM, which corresponds to the refp17 studied experimentally, was used as a template. The models were optimized with AMBER program^67^ and checked using PROCHECK^68^.

### Molecular Dynamics (MD)

MD simulations were performed with AMBER 12 software package^67^ and AMBER ff99SB force field. The system was solvated in a truncated octahedron periodic box by using TIP3P and counter ions were added to neutralize the system. The minimizations were performed keeping the position of protein restrained for tree minimization steps, by a force constant of 5000 Kcal/mol/Å² in the first, 100 Kcal/mol/Å² in the second, 10 Kcal/mol/Å² in the third and without protein restrained in the fourth. Next, the system was heated to 300 K using position restraints on entire protein with force constant of 10 kcal/mol/Å², from 0 K to 100 K at constant volume in 3 ns, from 100 to 300 at constant pressure in 2 ns.

Bond lengths involving hydrogens were constrained using the SHAKE algorithm and the equations of motion were integrated with a 2-fs time step. The non-bonded cutoff distance was 8Å and the Particle Mesh Ewald method was used to calculate long-range electrostatics interactions. The temperature of the system was regulated using the Langevin thermostat. Equilibration was reached in constant NPT ensemble for 7 ns. After equilibration, production MD was performed at 300 K using constant pressure without restraints for 500 ns. DSSP program was used to assign secondary structures^26^.

The convergence was evaluated calculating the time point beyond which the RMSD of the structure stayed in a range of 1 Angstrom around the centre of oscillations. The RMSD was calculated considering only the alpha carbon in the not flexible regions (RMSF < 3 Angstrom).

To assess the different conformations of p17s we performed cluster analysis using the default hierarchical agglomerative algorithm implemented in the cpptraj tool (AmberTools). The distance matrix was computed using RMS distance between each pair of frame. The dispersion of conformations along the dynamics within of the clusters was evaluated considering a dispersion index that takes into account the fraction of frames (*FR*_*i*_) and the average of RMSD within each cluster: ${\sum }_{i=1}^{Nc}RMS{D}_{i}\cdot (1-F{R}_{i})$, where *N*_*c*_ is the number of considered clusters.

The non-polar surfaces area was calculated using the software MDTraj^69^.

### CD spectroscopy and thermal denaturation

CD spectra of p17s at 2.5 μM in 10 mM PBS (pH 7.4) were obtained at room temperature on a Jasco J-810 spectropolarimeter using a 1-mm quartz cuvette. Protein thermal denaturation was carried out in PBS on the Jasco spectropolarimeter equipped with a temperature controller. 2.5 mL of protein solution (approximately 10 μM) prepared in PBS (pH 7.4) was aliquoted into a 3 mL cuvette. Under constant stirring, CD measurements at 222 nm were made at one degree interval between 25 °C and 90 °C, at a heating rate of 1 °C per minute. After each 1-minute heating, the solution in the cuvette waited for 20 s before signals were sampled over a 16 sec period. Heating and data acquisition were fully automated with control software provided by Jasco. Denaturation data were fitted to the following six-parameter equation derived from a two-state protein denaturation model as previously described^23, 70, 71^:

$${S}_{\theta }=\frac{({\alpha }_{n1}T+{\alpha }_{n2})+({\alpha }_{d1}T+{\alpha }_{d2}){e}^{\frac{{\rm{\Delta }}{H}_{m}}{RT}\frac{({T}_{m}-T)}{{T}_{m}}}}{1+{e}^{\frac{{\rm{\Delta }}{H}_{m}}{RT}\frac{({T}_{m}-T)}{{T}_{m}}}}$$

where Tm is melting temperature at which 50% of protein is denatured, ∆H_m_ is the enthalpy of denaturation, α_n1_, α_n2_, α_d1_, and α_d2_ are parameters for the parabolic equations defining native and denatured states, and the gas constant R = 8.314 J mol^−1^ K^−1^. The experiments for each protein were repeated three times.

### Dynamic light scattering

Concentrated protein solutions were filtered using a Whatman Anotop 10 filter (0.22 μm) and, after UV quantification, diluted in PBS to a final concentration of 10 μM each. Protein size measurements were carried out at 25 °C, pH 7.4, with a total ionic strength of 0.1 M, on a Malvern Zetasizer Nano ZS instrument. Twenty runs of 30 s each were taken and averaged, and the data were processed using the manufacturer provided software.

### NMR spectroscopy

NMR spectra were recorded at 25 °C on an 800 MHz (800.27 MHz for protons) Bruker Avance-series NMR spectrometer equipped with four frequency channels and a 5 mm triple-resonance z-axis gradient cryogenic probehead. A one-second relaxation delay was used and quadrature detection in the indirect dimensions was obtained with states-TPPI phase cycling; initial delays in the indirect dimensions were set to give zero- and first-order phase corrections of 90° and −180°, respectively^72, 73^. Data were processed using the processing program nmrPipe on Mac OS X workstations^74^. 2D NOESY experiments with a 150 ms mixing time were collected to monitor changes in the backbone and side-chain ^1^H protein resonances^75^. Typical NMR samples contained 3 mg/ml protein in a 10 mM sodium phosphate and 25 mM sodium chloride buffer (pH 7.4) to which 10% D_2_O (v/v) was added.

### Soft agar anchorage-independent growth assay

Raji cell clonogenic activity was evaluated in soft agar assays as previously described^18, 22, 23^.

### Western blot, high throughput phosphoproteomics and bioinformatics analysis

Raji cells, starved for 24 hours by serum deprivation, were stimulated or not with refp17, p17R76G and S75X (0.05, 0.1, 0.5 μg/ml) for 5 min, then lysed and prepared for Kinex^TM^ Antibody Microarray (KAM-850) according to manufacturer’s instructions (Kinexus Bioinformatics Corporation). Then, the samples were checked for pAkt and pPTEN levels by western blot analysis, as previously described^23^. In particular, the blots were incubated overnight at 4 °C with 1) mAb to pAkt (Cell Signaling Technology), 2) mAb to total Akt (Cell Signaling Technology), 3) mAb pPTEN (Ser380/Thr382/383) (Cell Signaling Technology), 3) mAb to GAPDH (glyceraldehyde-3-phosphate dehydrogenase). The lysates of cells stimulated with refp17, p17R76G and S75X (0.1 μg/ml) for 5 min were sent to Kinexus Bioinformatics Corporation for processing by Kinex™ KAM-850 Antibody Microarray, which contains 517 pan-specific and 337 phoshpo-site-specific probes, which recognize different epitopes on 466 proteins. The raw data, processed by Kinexus with the ImaGene software, were filtered according to company criteria. Only probes matching these criteria where considered for further analysis: signal to noise ratio >1.6; or signal to noise ratio <1.6 and noise inferior than the average noise of the array; maximum variation coefficient <0.2. The analysis was led with custom R, Visual Basic and MySQL procedures. Z-scores for normalization and z-ratios for comparisons were calculated as previously described^76^, according to the manufacturer’s recommendations, and a significativity threshold of 1.1 was adopted for the z-ratios. In order to rule out false positive array signals, a subsequent multi-immunoblotting verification step was performed according to the manufacturer’s recommendations. Therefore, on the 54 most relevant selected probes a pre-screening step was performed by Kinexus to select the reactive antibodies and rule out the false positive array signals due to antibody cross-reactivity. After pre-screening, 36 selected antibodies were used for a multi-immunoblotting analysis by Kinexus. Blot raw quantification data were processed by log transformation and normalization to either not treated or p17 treated samples for the interpretation of the results. STRING database was used to identify known and experimentally verified molecular interactions among proteins modulated by p17s^31^. Literature data mining was also performed by PubMed (http://www.ncbi.nlm.nih.gov/pubmed), PhosphoSite (http://www.phosphosite.org/), Uniprot (http://www.uniprot.org) and Genecards (http://www.genecards.org).

The modulated proteins were also used as input for the Cytoscape ClueGO functional analysis tool^50^ to cluster the proteins according to the KEGG pathways database.

### Statistical analysis

Data obtained from multiple independent experiments are expressed as the means ± the standard deviations (SD). The data were analyzed for statistical significance using one-way ANOVA. Bonferroni’s post-test was used to compare data. Differences were considered significant at *P* < 0.05. Statistical tests were performed using Prism 5 software (GraphPad).
